# Inactivation of Replication-Competent Vesicular Stomatitis Virus as SARS-CoV-2 Surrogate on Common Surfaces by Disinfectants

**DOI:** 10.3390/ijerph18147714

**Published:** 2021-07-20

**Authors:** Zachary C. Pope, Timothy J. Kottke, Aditya Shah, Richard G. Vile, Stacey A. Rizza

**Affiliations:** 1 Well Living Lab, Rochester, MN 55902, USA; 2 Department of Physiology and Biomedical Engineering, Mayo Clinic, Rochester, MN 55905, USA; 3 Department of Molecular Medicine, Mayo Clinic, Rochester, MN 55905, USA; kottke.timothy@mayo.edu (T.J.K.); vile.richard@mayo.edu (R.G.V.); 4 Division of Infectious Diseases, Mayo Clinic, Rochester, MN 55905, USA; shah.aditya@mayo.edu (A.S.); rizza.stacey@mayo.edu (S.A.R.)

**Keywords:** virology, disinfection, infection control, infectious diseases, COVID-19

## Abstract

Surface disinfection is part of a larger mitigation strategy to prevent the spread of the severe acute respiratory syndrome coronavirus-2 (SARS-CoV-2) virus causing coronavirus disease-2019 (COVID-19). Research evaluating the time, nature, and extent of surface disinfection of replication-competent viruses is needed. We evaluated the efficacy of two disinfectants against a replication-competent SARS-CoV-2 surrogate on three common public surfaces. Vesicular stomatitis virus expressing green fluorescent protein (VSV-GFP) was our replication-competent SARS-CoV-2 surrogate. Disinfection occurred using Super Sani-Cloth Germicidal Disposable Wipes and Oxivir Tb spray per manufacturer instructions to test the efficacy at reducing the presence, viability, and later replication of VSV-GFP on stainless steel, laminate wood, and porcelain surfaces using standardized methods after recovery and toxicity testing. During the main trials, we placed 100 µL spots of VSV-GFP at viral titers of 10^8^, 10^7^, and 10^6^ PFU/mL on each surface prior to disinfection. Trials were completed in triplicate and post-disinfection measurements on each surface were compared to the measurements of non-disinfected surfaces. Disinfectants were considered efficacious when ≥3-log_10_ reduction in the number of infectious VSV-GFP virus units was observed on a given surface during all trials. Both disinfectants produced a ≥3.23-log_10_ reduction in infectious VSV-GFP virus unit numbers, with all trials showing no viable, replication-competent VSV-GFP present on any tested surface. The two disinfectants eliminated the presence, viability, and later replication of VSV-GFP, our SARS-CoV-2 surrogate, on all surfaces. This information suggests that, if following manufacturer instructions, overcleaning surfaces with multiple disinfectant solutions may be unnecessary.

## 1. Introduction

Severe acute respiratory syndrome coronavirus 2 (SARS-CoV-2), the virus causing coronavirus disease 2019 (COVID-19), has resulted in high morbidity and mortality [1]. Evidence that most COVID-19 infections are transmitted by asymptomatic individuals [2,3] highlights the continued need for effective SARS-CoV-2 prevention and control measures. Indeed, given the time needed to distribute and administer approved vaccines at a global level, reducing SARS-CoV-2 transmission in public spaces continues to be crucial, with this research being also important for the control of seasonal respiratory illnesses such as influenza.

The Centers for Disease Control and Prevention (CDC) makes hazard control recommendations based on the Hierarchy of Controls [4]. This Hierarchy posits that the most effective hazard control measure is removal of the environmental hazard rather than relying solely on human behavior or personal protective equipment to mitigate risk. As impeding individuals from touching vulnerable tissues of the face after coming in contact with potentially virus-contaminated surfaces (i.e., fomites) remains challenging [5,6], effectively disinfecting high-touch surfaces (i.e., removing the environmental hazard) is imperative as part of a larger strategy to control the spread of respiratory illnesses such as COVID-19 and influenza. Completing disinfection experiments of viruses that are replication-competent is important given that replication-competent viruses contain the necessary genetic materials for virion synthesis and later propagation if contracted by a susceptible individual—a notable concern for viruses such as SARS-CoV-2 wherein replication-competent viral shedding can occur for over two weeks post infection [7,8]. Thus, if disinfectants are efficacious in reducing the presence and viability (i.e., whether the viral RNA components still present are infectious) of replication-competent viruses when used in simulated real-world scenarios and per manufacturer instructions, this serves as further verification of this manner of mitigation.

Although perspectives regarding how the SARS-CoV-2 virus spreads currently favor airborne transmission [9], disinfection of high-touch surfaces will remain an important component of a broader strategy to limit the spread of this virus and other seasonal respiratory illnesses. This is particularly true given that viable SARS-CoV-2 virus has been detected on surfaces such as stainless steel and plastic >72 h after placement on these surface types [10]. Therefore, data regarding the time, nature, and extent of disinfection required to disinfect replication-competent viruses on different surfaces are useful. As part of a larger, ongoing COVID-19 research program, we conducted disinfection experiments of a replication-competent SARS-CoV-2 surrogate virus to refine our methods before working with the replication-competent SARS-CoV-2 virus (experiments completed; manuscript in draft). Specifically, we examined the presence, viability, and subsequent replication of vesicular stomatitis virus expressing green fluorescent protein (VSV-GFP) post disinfection with two disinfectants on three common types of public surfaces: stainless steel, laminate wood, and porcelain. The VSV-GFP virus is a large, enveloped virus structurally similar to the SARS-CoV-2 virus and thus is a good SARS-CoV-2 surrogate. This virus surrogate approach has been used in prior research [11,12] regarding the disinfection of other coronaviruses given that investigations of coronaviruses require facilities with biosafety-level-3 (BSL-3) designations, and therefore, such research is more logistically challenging to conduct when quick dissemination of information is needed.

## 2. Materials and Methods

All trials were performed within a biosafety-level-2 (BSL-2) facility.

### 2.1. Cells and Viruses

We prepared VSV-GFP similar to that outlined in Kahn et al. [13]. Briefly, we seeded 41 × 15 cm plates with baby hamster kidney (BHK) cells maintained in Dulbecco’s minimum essential media (DMEM) + 5% fetal bovine serum (FBS) and grown in a humidified incubator set at 37 °C in 5% carbon dioxide until confluent. We then infected the cells with a multiplicity of infection (MOI; i.e., number of virions added to each cell; concentration of virus) of 0.01, determined by trypsinizing one of the 15 cm plates and counting how many cells were present per plate. Next, we diluted the virus stock in DMEM (no serum) to a final volume of 300 mL to ensure that 15 mL of 0.01 MOI diluted virus was present per 15 cm plate. Prior to infection of the BHK cells, we removed the medium and washed the cells with phosphate-buffered saline (PBS), after which we incubated it for one hour. Following incubation, we removed the virus medium and replaced it with 18 mL of DMEM and 10% FBS. We then incubated it until 100% cytopathic effects (CPE) were seen (approximately 18–24 h).

After removing the supernatant, we centrifuged the cells at 1200 rpm for 10 min in a Beckman centrifuge. We then filtered the supernatant through a 0.45 µM bottle-top filter and froze the supernatant until centrifuged on a sucrose cushion. Specifically, we pipetted 5 mL of 10% sucrose (10% *w/v* in PBS) into the bottom of a SW28 tube and slowly loaded 30 mL of the supernatant onto the top of the sucrose solution without disturbing the interface. Following this procedure, we centrifuged it at 27,000 rpm for one hour in a Beckman ultracentrifuge. We then removed the supernatant and sucrose after which we combined all pellets into 30 mL of PBS, loaded back on top of a 5 mL 10% sucrose cushion, and centrifuged again at 27,000 rpm for one hour. After this second ultracentrifuge cycle, we removed the PBS and sucrose, resuspended the pellets in 1 mL of PBS, aliquoted in 200 µL quantities, and froze at −70 °C.

### 2.2. Disinfectants

We employed two commonly used disinfectants, each with a different chemical composition and mode of application. The first disinfectant used was the Super Sani-Cloth^®^ Germicidal Disposable Wipe (PDI Healthcare; Woodcliff Lake, NJ, USA) [14]. This wipe is considered a hospital-grade disinfectant and comprises quaternary ammonium (0.25% by weight) and isopropyl alcohol (55.0% by weight), with the wipe being currently used to disinfect patient rooms by the institution at which this study was conducted. This disinfectant has a two-minute contact time. The second disinfectant employed was Oxivir Tb [15]. This disinfectant is hydrogen peroxide-based (0.5% by weight) and is commonly used by industry given its eco-friendly nature. Importantly, in contrast to the Super Sani-Cloth^®^ Germicidal Disposable Wipe, Oxivir Tb is a spray disinfectant and has a one-minute contact time.

### 2.3. Surfaces

We tested the efficacy of the disinfectants on three different surfaces—stainless steel (Alloy 304, T-300 Series; Penn Stainless Products, Quakertown, PA, USA), laminate wood (Mannington Laminate Wood; High Point, NC, USA), and porcelain (RENSTRAGRIS1224 Porcelain; Renaissance Tile & Bath, Chicago, IL, USA).

### 2.4. Recovery Experiments

Before the main disinfection experiments, we completed pilot work to ensure adequate recovery from each of the three surfaces to be tested. We started by seeding sixty 6-well plates with BHK cells at 1 × 10^8^ PFU/mL per well and incubating overnight at 37 °C. The next day, we spotted 100 µL of 10^8^, 10^7^, and 10^6^ PFU/mL of VSV-GFP onto autoclaved stainless steel, laminate wood, and porcelain surfaces, with 10 pieces of each surface type used. We then immediately swabbed the surfaces and placed swabs into tubes containing 500 µL PBS. Next, we pipetted 100 µL of each MOI into 10 tubes containing 500 µL PBS and placed the tubes on a 30 °C shaker for one hour. This was done to measure 100% recovery (without swabbing). We then conducted plaque assays on the PBS tubes. We started with a dilution of 10^2^ of the PBS solution and continued serially by 10 to 10^7^. We infected each well with 1 mL of diluted virus and allowed two hours for infection to occur at 37 °C. After this two-hour time period, we removed the virus and overlaid the wells with 2 mL of a 1:1 mixture of 6% CME and 2x DMEM, 20% FBS, and 2x pen/strep (P/S), with a 72 h incubation period following these procedures. At the conclusion of the 72 h incubation period, we removed the CME/MEM mixture, stained with crystal violet, washed the plates with water, dried, and counted the plaques formed. Results showed that at 10^8^ PFU/mL of VSV-GFP, we achieved a recovery of 35.0% and, for 10^7^ and 10^6^ PFU/mL, 2.4% and 1.1%, respectively. Given the low recovery percentages for the titers at 10^7^ and 10^6^ PFU/mL, we chose to only present the results of disinfectant efficacy on these three surface types using the 10^8^ PFU/mL titer despite also testing disinfectant efficacy at 10^7^ and 10^6^ PFU/mL (data available upon request). Indeed, efficaciousness at 10^8^ PFU/mL would allow for the reasonable assumption to be made that these disinfectants would be efficacious at lower titers (which the data not shown for the two lower titers demonstrates).

### 2.5. Toxicity Experiments

We also performed toxicity experiments on both disinfectants. Although the disinfectants are widely used, we chose to perform these experiments because we did not want to confuse the CPE that might be induced by the disinfectants with VSV-GFP-induced CPE. Six surfaces of each type were disinfected with each disinfectant (i.e., 12 surfaces for each surface type; six surfaces per disinfectant) without placing any VSV-GFP virus on these surfaces. For toxicity experiments with the Super Sani-Cloth^®^ Germicidal Disposable Wipe, we used one wipe and made two back and forth strokes to the surface being tested after which the surface was allowed the manufacturer-recommended two-minute contact time. At exactly two minutes, we swabbed the surface and transferred the swab into a tube containing 0.5 mL PBS. We then placed the tube into a shaker at 30 °C for one hour. Following this processing, we diluted PBS to 1:100 in DMEM and then added 1 mL of this solution into a single well and incubated it for one hour at 37 °C. We used this dilution given that it was the lowest dilution used in the plaque assays, 10^2^. Our reasoning was that if we did not see any toxicity at this dilution, it is unlikely that toxicity would be observed at higher dilutions. After this first incubation, we removed the solution and replaced it with 2 mL of normal medium. We then incubated it again for 72 h. At the end of the 72 h incubation period, we used a microscope to examine for toxicity and to take pictures followed by the removal of the medium and staining with crystal violet. Toxicity experiments with Oxivir Tb were performed identically, but the application method and dry time differed. Specifically, Oxivir Tb was sprayed upon each surface until wet, with the manufacturer-recommended one-minute dry time ensuing. After exactly one minute, the surface was swabbed and the methods outlined above were performed. Finally, it should be noted that we included a negative, non-disinfected control as part of our toxicity testing methodology to ensure the safety of these disinfectants when used in public spaces. These experiments confirmed the safety of both disinfectants when used in accordance with their manufacturer’s recommendations (data not shown).

### 2.6. Main Disinfection Experiments

The main disinfection experiments were performed within a fume hood. We have reported disinfection measurements at the viral titer of VSV-GFP in PFL/mL of 10^8^, with data for disinfection experiments done at titers of 10^7^ and 10^6^ available upon request. These titers are consistent with the range observed in prior coronavirus literature [16]. Prior to the disinfection trials with each surface type, we autoclaved the surfaces. The disinfection trials started with the seeding of 27 six-well plates with 1 × 10^6^ BHK cells and incubating overnight at 37 °C. We then diluted stock VSV-GFP (1 × 10^10^ PFU/mL) in PBS to obtain the titers to be experimentally tested. Specifically, we obtained 5 × 10^8^ PFU/mL through a 1:20 dilution of 100 µL VSV-GFP stock + 1900 µL PBS. The 5 × 10^7^ PFU/mL and 5 × 10^6^ PFU/mL through 1:10 dilutions of 150 µL VSV-GFP stock + 1350 µL PBS. Following these serial dilutions, we spotted nine surfaces of a given type with 100 µL of VSV-GFP at each titer. After spotting, we swabbed three surfaces immediately and placed these swabs into tubes containing 500 µL PBS for inclusion as our non-disinfected surfaces. The next three surfaces were disinfected with the Super Sani-Cloth^®^ Germicidal Disposable Wipe, with one wipe used and two back and forth strokes to the surface being tested. These surfaces were allowed the manufacturer-recommended two-minute dry time. At exactly two minutes, we swabbed the surface and transferred the swabs to a tube containing 500 µL of PBS. The final three surfaces were disinfected with Oxivir Tb, with the surfaces sprayed until visibly wet. Following the one-minute dry time, we swabbed these surfaces and placed the swabs into tubes containing 500 µL of PBS.

### 2.7. VSV-GFP Inactivation Determination via Plaque Assays

All PBS tubes containing the swabs referenced above were then placed on a 30 °C shaker for one hour after which a plaque assay was performed on these samples. We did this by diluting the virus PBS solutions serially from 10^2^ to 10^7^ and infecting confluent six-well plates of BHK cells with 1 mL of the diluted virus for one hour at 37 °C. The virus was then aspirated and overlaid with 2 mL CMC/MEM and then incubated for 72 h. Following the 72 h incubation period, we removed the CMC/MEM, washed with PBS, and stained with 0.5% crystal violet in 80% methanol. After washing off the stain and drying, plaques were counted.

From these plaque assays, we gleaned the necessary information to determine virus presence as well as replication and viability. Virus presence was determined by comparing the number of observed infected cells present on non-disinfected surfaces to those having undergone disinfection by the two disinfectants investigated. A post-disinfection PFU/mL value was used as a marker of replication and viability using the number of observed plaques and the dilution amounts employed and comparing this value to the PFU/mL value of non-disinfected surfaces. The following equation was used to calculate these values: PFU/mL = No. of plaques x dilution/1.0 mL. The disinfectant was considered efficacious when a ≥3-log base 10 (log_10_) reduction occurred in the number of infectious VSV-GFP virus units on a given surface during all trials (i.e., ≥99.9% reduction), calculated in Microsoft Excel using the LOG10 function.

## 3. Results

The raw plaque count data and the average PFU/mL of virus recovered on each surface type with and without disinfection are shown in Table 1, Table 2 and Table 3 for stainless steel, laminate wood, and porcelain, respectively. On the disinfected surfaces, no plaques were present post disinfection on any surface after 72 h of incubation for either the Super Sani-Cloth^®^ Germicidal Disposable Wipe or Oxivir Tb when used per manufacturer recommendations. Disinfection by both disinfectants at each MOI on all surface types contributed to ≥3-log_10_ reduction in viable VSV-GFP virus (see Table 1, Table 2 and Table 3; ≥99.9% reduction).

## 4. Discussion

The enveloped nature of the VSV-GFP virus makes it a good SARS-CoV-2 surrogate. Our observations confirmed that these two widely used disinfectants are highly efficacious at reducing the presence, viability, and later replication of the VSV-GFP virus on stainless steel, laminate wood, and porcelain when used per manufacturer instructions. While we are currently completing work with the SARS-CoV-2 virus, it is likely that these disinfectants may help limit any fomite-based COVID-19 infections on most similar hard nonporous surfaces in public spaces as more individuals move back into these spaces.

Our observations are comparable to past coronavirus surrogate research. Hulkower et al. [11] examined the efficacy of several healthcare germicidal solutions in the reduction of mouse hepatitis virus and transmissible gastroenteritis virus, as surrogates for SARS-CoV, on stainless steel surfaces following a one-minute contact time. They observed that only 70% ethanol was efficacious at producing a greater than ≥3-log_10_ reduction for both viruses within one minute; yet the authors set the contact time at one minute and did not follow manufacturer-recommended contact times. In another study by Dellanno et al. [12] that had greater methodological alignment with our trials, researchers used common household disinfectants and antiseptics per manufacturer instructions to disinfect murine hepatitis virus on surfaces. They observed all disinfectants and antiseptics to be efficacious at producing ≥3-log_10_ reduction in the presence of this virus when used per manufacturer instructions, these products comprising (1) 0.10% alkyl dimethyl benzyl ammonium saccharinate with 79% ethanol; (2) 0.12% PCMX; (3) 0.21% sodium hypochlorite; (4) 0.05% triclosan; and (5) 0.23% pine oil. Importantly, investigations of disinfectant efficacy against select coronaviruses have also demonstrated results similar to our trials. Kariwa et al. [17] observed results akin to Hulkower et al. [11], with 70% ethanol reducing SARS-CoV infectivity to below detectable levels—equivalent to that of povidone iodine products commonly used for disinfection purposes and tested within their study. Omidbakhsh et al. [18] noted that the hydrogen peroxide-based ACCEL Tb, a disinfectant having the same chemical composition and manufacturer as Oxivir Tb, resulted in a >4-log_10_ reduction in human coronavirus 229E. The current investigation adds to this literature, showing that a disinfectant comprising 0.25% quaternary ammonium and 55.0% isopropyl alcohol (Super Sani-Cloth^®^ Germicidal Disposable Wipes) can satisfactorily reduce the presence and viability of a replication-competent virus on three different nonporous hard surfaces, with 0.50% hydrogen peroxide solution (Oxivir Tb) again demonstrating that it can do the same, as in Omidbakhsh et al. [18]

Current evidence on the spread of SARS-CoV-2 has suggested that airborne transmission likely accounts for most COVID-19 infections [9,19,20]. Yet, the disinfection of high-touch surfaces is still part of a larger set of precautions (e.g., mask wear, physical distancing, handwashing) to limit the spread of the SARS-CoV-2 virus and other respiratory illnesses. From this perspective, our observations are important and align with the CDC’s hazard control recommendations based on the Hierarchy of Controls [4]. That said, two limitations should be acknowledged when interpreting our results. First, the inoculums of the virus used during the current investigation were higher than what would be expected within the real-world environment. This was due to the need to achieve adequate recovery prior to performing our assay procedures. Despite this limitation, our study suggested that basic manufacturer-recommended disinfection procedures are sufficient for the disinfection of high inoculums of a virus and that perhaps large amounts of time and resources need not be diverted to expensive surface disinfection methods. Second, only hard, nonporous surfaces were used during these trials. While we may investigate softer, more porous surfaces in the future, other investigators are encouraged to examine disinfection of these other surfaces too.

## 5. Conclusions

Although the importance of disinfecting surfaces continues to be debated, high-touch surface disinfection is still an important mitigation component in which COVID-19 infections are limited. We demonstrated the safety and efficacy of two widely used and available disinfectants at eliminating the presence, replication, and viability of VSV-GFP, as a SARS-CoV-2 surrogate, on stainless steel, laminate wood, and porcelain when used per manufacturer instructions. Given the structural similarity between VSV-GFP and SARS-CoV-2, we believe that our ongoing work with the latter virus will demonstrate similar results. Therefore, as considerable time and effort have been allocated for garnering resources for advanced surface disinfection measures, our results suggest that these measures may be unnecessary and may be contributing to overutilization of sparse and already stretched resources.

## Figures and Tables

**Table 1:** Disinfection data for VSV-GFP 100 µL spot on stainless steel.

		Plaques Counted						Measured Avg. PFU/mL	Log Reduction ≥ 3 Post Disinfection?
MOI	Disinfectant	10^2^	10^3^	10^4^	10^5^	10^6^	10^7^		
**10^8^**	*None*	>	>	>	>	15	1	2.77 × 10^7^	***Log_10_ Value: 7.44***
	*None*	>	>	>	>	18	2		
	*None*	>	>	>	>	50	4		
	*Super Sani-Cloth Wipes*	0	0	0	0	0	0	0	Yes
	*Super Sani-Cloth Wipes*	0	0	0	0	0	0		
	*Super Sani-Cloth Wipes*	0	0	0	0	0	0		
	*Oxivir Tb Spray*	0	0	0	0	0	0	0	Yes
	*Oxivir Tb Spray*	0	0	0	0	0	0		
	*Oxivir Tb Spray*	0	0	0	0	0	0		

Notes: MOI = multiplicity of infection (i.e., number of virions added to each cell; concentration of virus); PFU = plaque forming units (i.e., number of infectious units). > = above the limit of detection.

**Table 2:** Disinfection data for VSV-GFP 100 µL spot on laminate wood.

		Plaques Counted						Measured Avg. PFU/mL	Log Reduction ≥ 3 Post Disinfection?
MOI	Disinfectant	10^2^	10^3^	10^4^	10^5^	10^6^	10^7^		
**10^8^**	*None*	>	>	>	>	120	10	9.83 × 10^7^	***Log_10_ Value: 7.99***
	*None*	>	>	>	>	71	7		
	*None*	>	>	>	>	104	9		
	*Super Sani-Cloth Wipes*	0	0	0	0	0	0	0	Yes
	*Super Sani-Cloth Wipes*	0	0	0	0	0	0		
	*Super Sani-Cloth Wipes*	0	0	0	0	0	0		
	*Oxivir Tb Spray*	0	0	0	0	0	0	0	Yes
	*Oxivir Tb Spray*	0	0	0	0	0	0		
	*Oxivir Tb Spray*	0	0	0	0	0	0		

Notes: MOI = multiplicity of infection (i.e., number of virions added to each cell; concentration of virus); PFU = plaque forming units (i.e., number of infectious units). > = above the limit of detection.

**Table 3:** Disinfection data for VSV-GFP 100 µL spot on porcelain.

		Plaques Counted						Measured Avg. PFU/mL	Log Reduction ≥ 3 Post Disinfection?
MOI	Disinfectant	10^2^	10^3^	10^4^	10^5^	10^6^	10^7^		
**10^8^**	*None*	>	>	>	24	2	0	2.70 × 10^6^	***Log_10_ Value: 6.43***
	*None*	>	>	151	14	1	0		
	*None*	>	>	>	42	4	0		
	*Super Sani-Cloth Wipes*	0	0	0	0	0	0	0	Yes
	*Super Sani-Cloth Wipes*	0	0	0	0	0	0		
	*Super Sani-Cloth Wipes*	0	0	0	0	0	0		
	*Oxivir Tb Spray*	0	0	0	0	0	0	0	Yes
	*Oxivir Tb Spray*	0	0	0	0	0	0		
	*Oxivir Tb Spray*	0	0	0	0	0	0		

Notes: MOI = multiplicity of infection (i.e., number of virions added to each cell; concentration of virus); PFU = plaque forming units (i.e., number of infectious units). > = above the limit of detection.

## Data Availability

The data presented in this study are available on request from the corresponding author. The data are not publicly available due to privacy concerns.
